# Recent Understanding in the Chemical Vapor Deposition of Multilayer Graphene: Controlling Uniformity, Thickness, and Stacking Configuration

**DOI:** 10.3390/nano13152217

**Published:** 2023-07-30

**Authors:** Hyo Chan Hong, Jeong In Ryu, Hyo Chan Lee

**Affiliations:** Department of Chemical Engineering, Myongji University, Yongin 17058, Republic of Korea

**Keywords:** multilayer graphene, chemical vapor deposition, uniformity, thickness, stacking

## Abstract

Multilayer graphene has attracted significant attention because its physical properties can be tuned by stacking its layers in a particular configuration. To apply the intriguing properties of multilayer graphene in various optoelectronic or spintronic devices, it is essential to develop a synthetic method that enables the control of the stacking configuration. This review article presents the recent progress in the synthesis of multilayer graphene by chemical vapor deposition (CVD). First, we discuss the CVD of multilayer graphene, utilizing the precipitation or segregation of carbon atoms from metal catalysts with high carbon solubility. Subsequently, we present novel CVD approaches to yield uniform and thickness-controlled multilayer graphene, which goes beyond the conventional precipitation or segregation methods. Finally, we introduce the latest studies on the control of stacking configurations in bilayer graphene during CVD processes.

## 1. Introduction

Graphene is a honeycomb-lattice material composed of *sp*^2^-bonded carbon atoms. Graphene is of interest owing to its excellent properties such as its thermal conductivity and electron mobility [1,2,3,4,5,6,7,8]. Unique physical properties of graphene can emerge by stacking graphene layers in a particular configuration (the number of layers and the twist angle between adjacent graphene layers). Monolayer graphene is a gapless semimetal, whereas AB-stacked bilayer graphene has a tunable bandgap [9,10]. On the other hand, turbostratic multilayer graphene, characterized by disordered stacking of its layers, possesses exceptional electrical properties [11]. The weak interlayer coupling in turbostratic multilayer graphene results in a high charge carrier mobility comparable to that of monolayer graphene. As a result, turbostratic multilayer graphene exhibits excellent electrical conductivity [12,13,14]. Recently, superconductivity has been demonstrated for twisted bilayer graphene whose twist angle is near to 1.1°, referred to as the “magic angle” [15,16,17,18].

To utilize the intriguing properties of multilayer graphene for various optoelectronic or spintronic devices, the development of a synthesis method for multilayer graphene with a controlled stacking configuration is essential. There has been significant progress in the chemical vapor deposition (CVD) of large-area graphene [19,20,21,22,23,24,25,26,27]. However, it is still challenging to synthesize uniform multilayer graphene with controlled stacking configurations. In this review article, we first discuss the current understanding of the CVD of multilayer graphene, with a focus on the precipitation or segregation of carbon atoms from catalysts with high carbon solubility. This will be followed by recent CVD approaches that aim to achieve uniform and thickness-controlled multilayer graphene, beyond the traditional precipitation or segregation methods. We then review the latest studies on the control of the stacking configuration of bilayer graphene during CVD processes. Finally, we provide a summary and discuss the future prospects for the CVD of multilayer graphene.

## 2. CVD of Multilayer Graphene by a Precipitation/Segregation Mechanism

### 2.1. Ni Catalysts

Both Cu and Ni catalysts have been widely used for the CVD of graphene [28,29,30,31,32,33,34,35,36,37,38]. However, the growth mechanism of graphene on each catalyst is different. Cu has negligibly low carbon solubility. Thus, carbon atoms generated from the dehydrogenation of carbon sources (usually methane) immediately participate in a surface-mediated reaction on the Cu surface, forming graphene. When the Cu surface is fully covered by a graphene layer, the Cu surface loses its catalytic activity because carbon sources cannot adsorb on the surface. As a result, highly uniform monolayer graphene is obtained on the Cu surface. On the other hand, the carbon solubility of Ni catalysts is very high. Thus, dehydrogenated carbon sources are dissolved inside the Ni catalyst during the CVD process. The dissolved carbon atoms are then precipitated or segregated during the cooling stage [39].

In contrast with Cu catalysts, multilayer graphene can be easily synthesized using Ni catalysts. When a large number of carbon atoms are dissolved during the annealing process, a large number of carbon atoms participate in forming graphene, yielding multilayer graphene. However, it is challenging to control the number of graphene layers and obtain uniform multilayer graphene using Ni catalysts because the precipitation or segregation of carbon atoms during the cooling stage is a very rapid process. Thus, extensive studies have been carried out to optimize the cooling rate, gas flow rates, and annealing temperature to control the thickness of the graphene and enhance the uniformity of the multilayer graphene [40,41,42,43,44,45]. 

The polycrystallinity of Ni catalysts also degrades the uniformity of multilayer graphene. Polycrystalline Ni catalysts contain numerous grain boundaries where segregation and precipitation of C atoms are particularly favored compared to the surface of large Ni grains. As a result, significant nonuniformity in graphene usually occurs at the grain boundaries of Ni catalysts. In addition, owing to the different crystallographic orientations of Ni grains in Ni catalysts, the orientations of graphene nuclei on the Ni catalysts may be randomly distributed, resulting in polycrystalline graphene with small grains [46].

### 2.2. Cu/Ni Alloys

To overcome the limitations of using Ni catalysts in terms of the uniformity of graphene, Cu/Ni alloys have been evaluated as catalysts for thickness-controlled multilayer graphene [47]. By combining Cu and Ni in an alloy catalyst, it is possible to achieve a catalyst with improved performance compared to either pure Cu or Ni catalysts. The uniformity of graphene can be improved with the aid of the Cu content, while the thickness of graphene can be controlled owing to the Ni content in the alloy catalyst.

**Figure 1:**
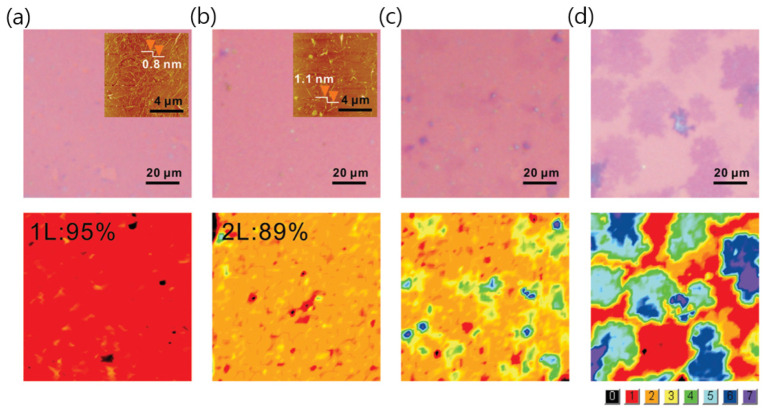
Morphologies and layer distributions of various few-layer graphene samples segregated from a Cu/Ni alloy at 900 °C after being transferred to a 300 nm SiO_2_/Si substrate. The upper part shows optical microscopy (OM) images of graphene samples corresponding to the different Ni atomic percentages in the alloy: (**a**) 5.5%, (**b**) 10.4%, (**c**) 18.9%, and (**d**) 100%. The insets in (**a**,**b**) show the corresponding atomic force microscopy (AFM) images. The lower part presents layer distributions determined by a red–green–blue (RGB) color analysis of the OM images. Different colors in the bottom right corner indicate the layer thicknesses according to the provided color code. Reproduced from [47]. Copyright 2011, American Chemical Society.

Early studies on the use of Cu/Ni alloys focused on the optimization of the content of Ni in the alloy (Figure 1) [47]. The optimized concentration of Ni in Cu/Ni alloys is different from laboratory to laboratory [48]. However, generally, monolayer graphene is mainly formed on a Cu/Ni alloy when the content of Ni is considerably lower than 10%, which implies that the Cu/Ni alloy behaves as a pure Cu catalyst. As the Ni concentration is far above 15%, thick multilayer graphene is formed, as with a pure Ni catalyst. Notably, the use of the Cu/Ni alloy provides uniform bilayer graphene with high coverage when the Ni concentration is appropriately optimized.

To further improve the uniformity of multilayer graphene grown on a Cu/Ni alloy, the CVD of graphene on a single-crystal Cu/Ni alloy [49] was investigated. In the pioneering research, a single-crystal Cu/Ni(111) alloy was fabricated by depositing a Cu thin film on a single-crystal sapphire substrate, and, subsequently, a Ni thin film [50]. Owing to the high processing temperature during CVD, Cu and Ni diffuse into each other and form a single-crystal Cu/Ni(111). On this catalyst, uniform bilayer graphene with a coverage of 93% was achieved. 

In 2020, Ruoff et al. prepared an extremely large single-crystal Cu/Ni(111) foil as a catalyst for large-area uniform multilayer graphene [27]. First, a large-area single-crystal Cu(111) foil was prepared using the contact-free annealing method [51]. By minimizing contact stresses, the colossal grain growth of a Cu foil can be achieved. To fabricate a single-crystal Cu/Ni(111) alloy, a Ni layer was plated on both sides of the prepared large-area Cu(111) foils, and then the foils were annealed at high temperature (1050 °C) for several hours. As a result, a large-area single-crystal Cu/Ni(111) alloy foil (3 cm × 5 cm) was successfully prepared. Owing to the single-crystalline catalysts on a large scale, the synthesized graphene exhibited an extremely high uniformity on a large scale. The thermal conductivity of the synthesized AB-stacked bilayer graphene was 2300 W m^−1^ K^−1^, comparable to that of mechanically exfoliated bilayer graphene (~2800 W m^−1^ K^−1^), demonstrating the high quality of the synthesized AB-stacked bilayer graphene. In addition, the thickness of the graphene was successfully controlled by tuning the content of Ni in the alloy catalyst.

## 3. CVD of Multilayer Graphene by Other Mechanisms

### 3.1. Bulk Diffusion of Carbon Atoms

The graphene growth on the Cu surface is surface mediated, and thus uniform graphene is obtained on the Cu surface. Therefore, uniform multilayer graphene can be obtained once the self-limiting characteristics of the Cu surface are eliminated.

**Figure 2:**
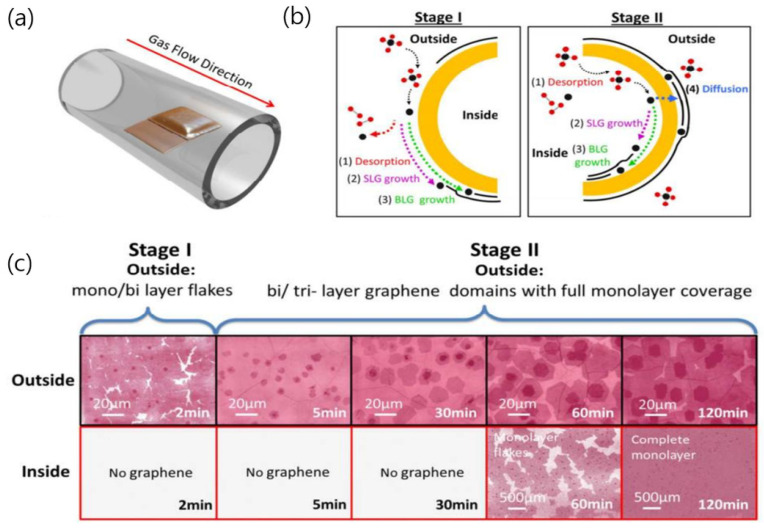
(**a**) Illustration of a sample grown in parallel in a quartz tube, showing both flat and enclosure regions. (**b**) Growth mechanism of the bilayer graphene on the outside surface: stage I depicts the growth when the monolayer graphene is incomplete, while stage II represents the growth after the completion of the monolayer graphene at the outside surface. (**c**) Time-dependent graphene growth on both sides of the Cu enclosure. Graphene is transferred onto the SiO_2_/Si substrate for improved imaging, with different shades of pink representing the varying thicknesses of graphene. On the inside surface, after 60 min, graphene monolayer flakes with bi/trilayer regions are observed. After 120 min, the monolayer graphene film growth is completed on the inside, while the bilayer growth on the outside seems to reach saturation. The graphene growth process is categorized into two stages based on the completeness of the monolayer growth on the outside surface of the Cu enclosure. Reproduced with permission from [35]. Copyright 2014, American Chemical Society.

In 2014, Kong et al. investigated the growth of graphene using Cu pockets (Figure 2a) [35]. Notably, multilayer graphene was preferentially formed on the outside surface of Cu pockets while monolayer graphene was synthesized on the inside surface (Figure 2b). These characteristic results were attributed to the bulk diffusion of C atoms from the inside surface to the outside surface of Cu pockets. Because the flux of CH_4_ gases into the inside of Cu pockets is limited, the growth rate of graphene on the inside surface of Cu pockets was considerably lower than that on the outside of the Cu pockets. Therefore, the inside Cu surface can be still open to the adsorption and dehydrogenation of CH_4_ molecules whereas graphene fully covers the outside Cu surface of the Cu pockets. This leads to asymmetry in the carbon concentration inside the bulk Cu: the carbon concentration is higher near the inner Cu surface. As a result, there is a bulk diffusion of C atoms from the inside surface to the outside surface of Cu pockets, and multilayer graphene can form on the outside even after the monolayer graphene has fully covered the outside Cu surface (Figure 2c). The uniformity of the multilayer graphene grown on Cu pockets can be further improved by introducing a mild oxidation pretreatment step for Cu pockets prior to graphene growth [52]. Compared to the non-treated Cu surface, it is energetically more favorable for C atoms to extend the second-layer graphene growth, rather than nucleation for the third-layer graphene on the oxidized Cu surface. Therefore, the Frank–van der Merwe growth of bilayer graphene was observed on the pretreated Cu pockets whereas the growth of the bilayer graphene on the bare Cu pockets followed the Stranski–Krastanov growth mode.

**Figure 3:**
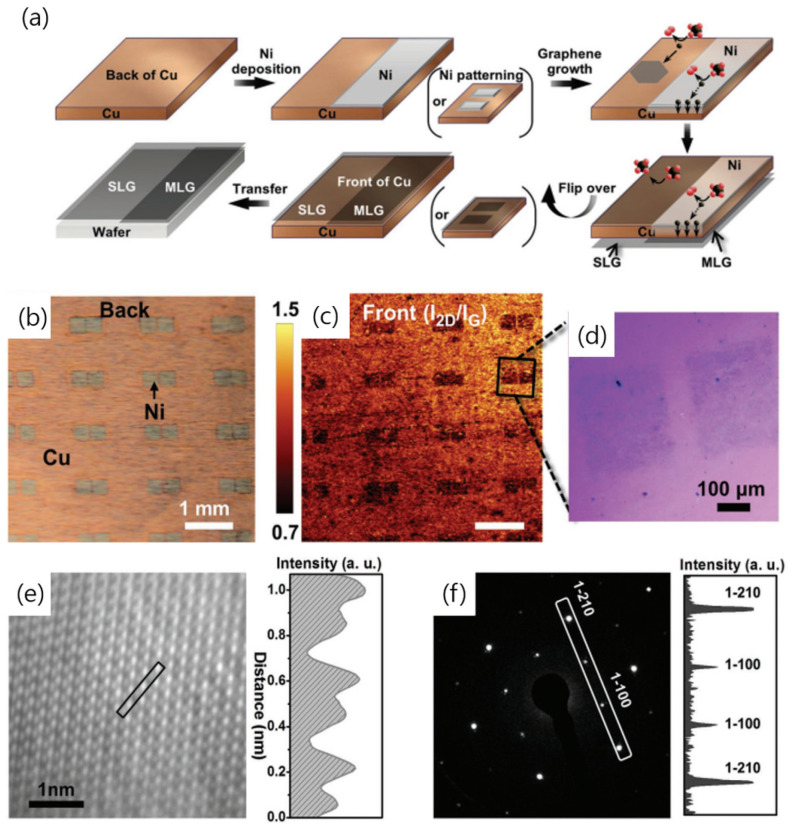
(**a**) Schematic of the growth of graphene on a Cu foil, where half of the back is covered by a Ni film or a Ni pattern. (**b**) Photograph showing the Ni-patterned back of the Cu/Ni structure. (**c**) Map of the Raman *I*_2D_/*I*_G_ ratio. (**d**) Photograph showing graphene grown on the front side of the patterned Cu/Ni foil, subsequently transferred onto a SiO_2_ substrate. (**e**) Filtered high-resolution transmission electron microscopy (HR-TEM) image of graphene (left) accompanied by the intensity profile obtained from the image (right). (**f**) Selected-area electron diffraction (SAED) pattern (left) and intensity profile derived from the SAED pattern (right). Reproduced with permission from ref [53]. Copyright 2017, WILEY-VCH Verlag GmbH & Co. KGaA, Weinheim, Germany.

For the Cu pockets, the bulk diffusion of C atoms is induced by asymmetry in the growth rate of graphene in and outside of the Cu pockets. In 2017, another strategy to induce the bulk diffusion of C atoms inside Cu was reported [53]. To this end, a 200 nm-thick Ni film was deposited on the back side of Cu foils. During the CVD growth, the back side of the Cu foil forms a Cu/Ni alloy with high Ni content due to the deposited Ni thin film whereas the front side of the Cu foil remains a pure Cu surface (Figure 3a). On the front side, a negligible amount of C atoms is dissolved during the CVD process. On the other hand, the high content of Ni on the back side results in dissolution of C atoms during CVD, causing bulk diffusion of C atoms to the front side due to the C concentration gradients. As a result, uniform and multilayer graphene can form on the front side of the Cu foil (Figure 3b–d). The synthesized graphene exhibited a low sheet resistance of 50 Ω sq^−1^ with a transmittance of 90% after the doping process. In addition, the thickness of the multilayer graphene can be easily tuned simply by controlling the thickness of the depositing Ni thin films. 

### 3.2. Intentional Formation of Vacancies in Graphene: Cu–P and Cu–S Alloys

**Figure 4:**
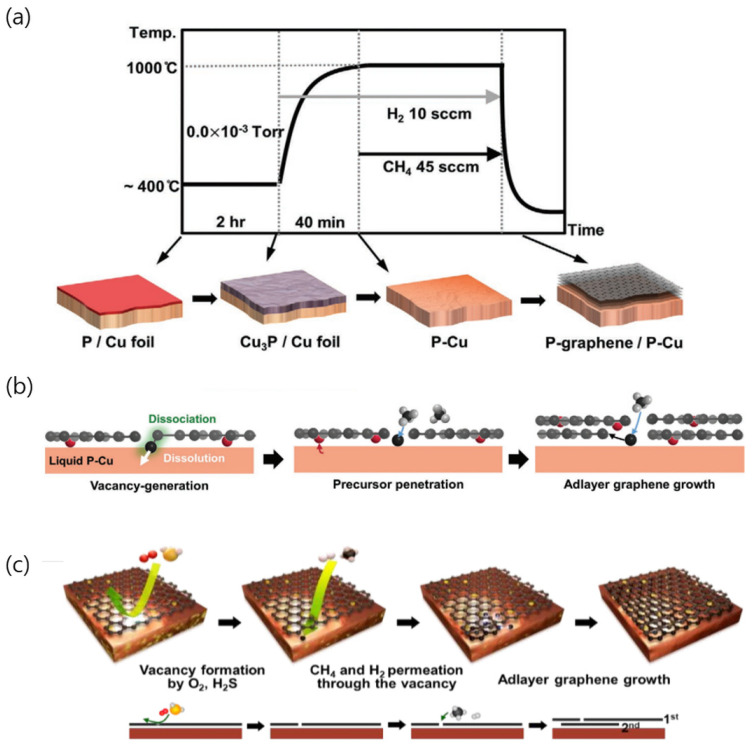
(**a**) Schematic of graphene growth. (**b**) Schematic of the growth mechanism and doping of the multilayer graphene. Reproduced with permission from [54]. Copyright 2020, Wiley-VCH GmbH. (**c**) Schematic of the proposed mechanism of growth of the multilayer graphene and S doping. Reproduced with permission from [55]. Copyright 2020, American Chemical Society.

The Cu–P and Cu–S alloy systems result in the formation of multilayer graphene [54,55]. In both cases, vacancies are generated in the early formed first-layer graphene during CVD. In the case of the Cu–P alloy, the system forms a eutectic solid solution. Thus, the surface of the catalyst is in a liquid phase during the CVD process (Figure 4a). The mobile Cu atoms of the catalyst result in vacancies in the graphene. On the other hand, in the case of the Cu–S alloy, sulfur atoms that have been dissolved on the Cu surface are segregated or precipitated from Cu and react with the H_2_ gas, forming an H_2_S gas. The H_2_S gas etches the early formed graphene layer on the surface. Once vacancies are formed in the first-layer graphene, carbon sources such as methane can easily penetrate the first-layer graphene and adsorb on the Cu surface. Consequently, graphene adlayers can form between the early formed graphene and Cu surface (Figure 4b).

Owing to the growth mechanism of the multilayer graphene on the Cu–P and Cu–S alloy systems, the resultant multilayer graphene inherently contains some defects. However, the use of Cu–P or Cu–S alloy systems has two advantages over other catalysts for multilayer graphene. The patterned doping of P atoms or S atoms on the Cu surface is very easy. The patterned doping of the Cu surface directly yields the patterned growth of multilayer graphene. In addition, during the CVD process, P or S atoms adsorb on the graphene surface, yielding doped graphene (Figure 4c). The degree of doping can be tuned by controlling the gas flow rate of H_2_. 

## 4. Control of the Stacking Configuration of Bilayer Graphene

### 4.1. AB-Stacked Bilayer Graphene

Various properties of the bilayer graphene are determined by the stacking configuration of the two graphene layers. In this regard, extensive studies have been carried out to develop a method to control the twist angle of bilayer graphene during the synthesis stage. Despite significant progress in this field, precise control of the stacking configuration of bilayer graphene is challenging. In this review, the recent understanding on the mechanism that determines the twist angle of bilayer graphene during CVD is introduced when Cu or Cu/Ni alloys are used as catalysts.

Bernal-stacked bilayer graphene in a transverse electric field exhibits a band gap whereas monolayer graphene is a gapless material. Therefore, numerous studies have focused on the production of AB-stacked bilayer graphene using Cu or Ni catalysts [56].

#### 4.1.1. Cu

**Figure 5:**
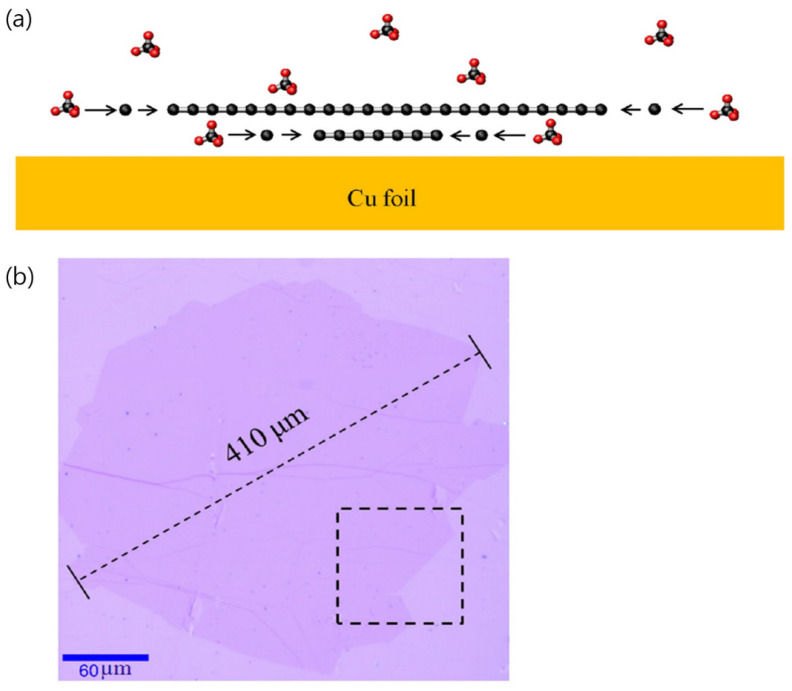
(**a**) Schematic of the growth mechanism of the adlayer graphene on a Cu substrate, showcasing submillimeter bilayer graphene. (**b**) Optical image of a transferred graphene grain on a 300 nm SiO_2_/Si substrate. Reproduced with permission from [32]. Copyright 2013, American Chemical Society.

On Cu catalysts, Li et al. conducted a carbon isotope labeling study and revealed that adlayer graphene grows below the first-layer graphene [32]. When the Cu surface is not fully covered by the first-layer graphene so that the Cu surface has catalytic activity for the dehydrogenation of carbon sources, dehydrogenated carbon atoms can intercalate between the first-layer graphene and Cu surface. It is also possible that carbon sources such as CH_4_ directly diffuse into the gap between the first-layer graphene and Cu surface. The adlayer graphene can then form below the first-layer graphene with the aid of the Cu surface (Figure 5a). This growth mechanism of the adlayer on the Cu surface suggests that the coverage of the bilayer graphene can be increased by reducing the growth rate of the first-layer graphene. When the pressure of H_2_ was increased to promote hydrogen-assisted etching of the edge of the first-layer graphene, which slows the growth of the first-layer graphene, the formation of single-crystal bilayer graphene with a grain size of 410 μm (Figure 5b) was demonstrated [32]. In addition to the high pressure of H_2_, other methods to etch the first-layer graphene and thus slow the growth of the first-layer graphene can be used to synthesize large-area bilayer graphene [57]. Notably, a large portion of the bilayer graphene synthesized in this manner exhibits a Bernal-stacking configuration. This implies that the provision of a sufficient time for the graphene adlayers to find an energetically favorable twist angle during the CVD process is important to form AB-stacked bilayer graphene.

#### 4.1.2. Cu/Ni Alloys

**Figure 6:**
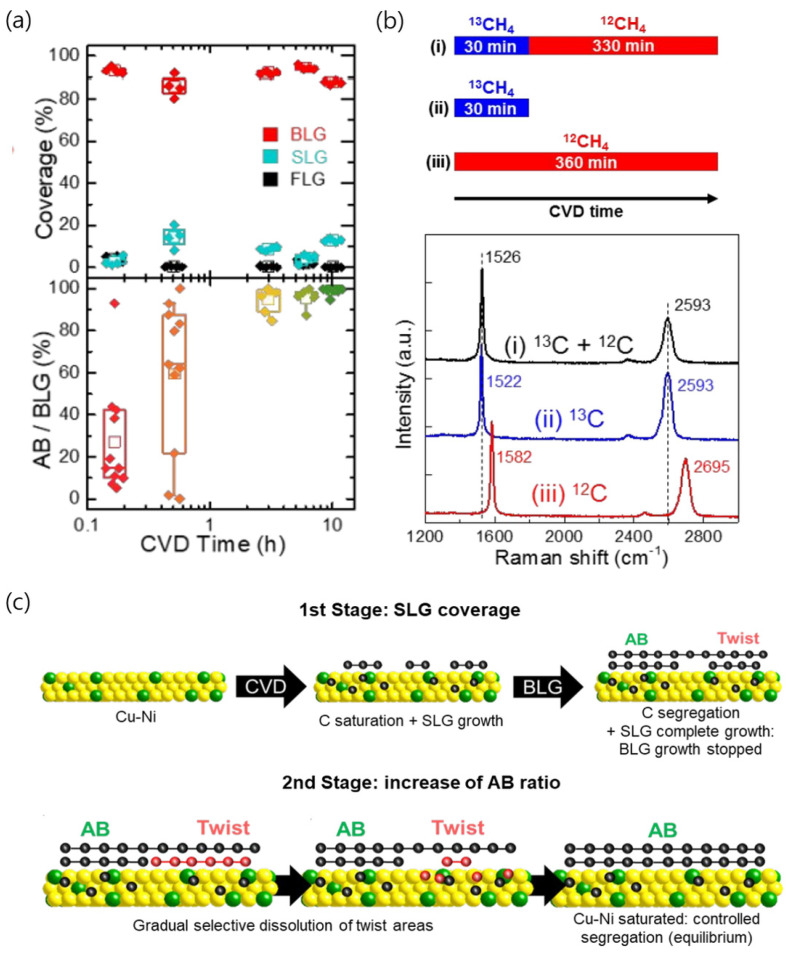
(**a**) (Upper) Evolution of surface coverages for single-layer graphene (SLG), bilayer graphene (BLG), and few-layer graphene (FLG) according to the CVD time. (Lower) Temporal changes in the AB stacking ratio of the BLG. In both graphs, the mean values are represented by open squares, while the boxes indicate the ranges between the 25th and 75th percentiles. (**b**) (Upper) Isotope labeling was employed during the growth of the BLG to provide insights into its growth dynamics. The reaction profiles utilized in the isotope labeling CVD processes are presented. (Lower) Representative Raman spectra of an AB-stacked BLG obtained under the conditions indicated in the upper section. (**c**) Mechanism underlying the formation of the uniformly AB stacked BLG on a Cu/Ni alloy catalyst. The BLG growth progresses until the entire surface of the Cu/Ni substrate is completely coated with graphene. The evolution of the stacking order is observed, with the AB ratio increasing over time during CVD due to the selective dissolution of the twisted bilayer graphene (T-BLG) and subsequent segregation from the Cu/Ni surface, which reaches saturation. Reproduced with permission from [56]. Copyright 2020, American Chemical Society.

Cu/Ni alloys yield highly uniform AB-stacked bilayer graphene under optimized growth conditions. AB-stacked bilayer graphene with a yield of 99.4% was achieved on sputtered Cu/Ni alloy thin films [56]. Graphene grown on a single-crystal Cu/Ni(111) alloy foil with a Ni concentration of 16.6 at.% yields AB-stacked bilayer graphene with a yield of ~100% [27]. Ago et al. proposed a growth mechanism of AB-stacked bilayer graphene on Cu/Ni alloy surfaces with a high yield [56]. The AB stacking ratio of the bilayer graphene on Cu/Ni alloy thin films strongly depended on the growth period. A short growth period (10 min) resulted in bilayer graphene with an AB stacking ratio of only 27%. However, as the growth period was extended to 3 h, the AB stacking ratio increased largely (>90%). The AB stacking ratio increased to 99.4% after 10 h of growth (Figure 6a). Notably, according to the isotope labeling of the growth of BLG, no new carbon atoms were incorporated from the gas phase after the first 30 min of growth (Figure 6b). This observation suggests that the observed structural reconstruction of the bilayer graphene and increase in the AB stacking ratio were not due to the exchange of carbon atoms from the gas phase. Instead, it was proposed that the structural reconstruction of the bilayer graphene and the increase in the AB stacking ratio were achieved through carbon dissolution–segregation processes, which were assisted by the catalyst. The results suggested that the preferential dissolution of the energetically unfavorable twisted bilayer graphene in the Cu/Ni alloy was followed by the segregation/precipitation of carbon atoms from the saturated Cu/Ni alloys, leading to the formation of the stable AB-stacked bilayer graphene (Figure 6c).

Recently, using in-situ low-energy electron diffraction, the growth dynamics of bilayer graphene on Ni(111) have been investigated [58]. The formation of twisted second layers was more kinetically favorable than when aligned at high temperatures (>790 °C), while the opposite behavior was observed at lower temperatures (<720 °C). The first graphene layers were aligned with the Ni(111) surfaces and segregated at 850 °C through a process limited by thermodynamics. The second layers were then grown at the decreased temperature, which resulted in the formation of an extremely uniform 15°-twisted BLG at 790 °C and an AB-stacked BLG at 720 °C. The stacking configuration of the bilayer graphene is significantly influenced by the growth temperature, which determines whether the growth of the second layer is kinetically (>790 °C) or thermodynamically (<720 °C) limited.

### 4.2. Twisted Bilayer Graphene

Non-Bernal-stacked bilayer graphene, or twisted bilayer graphene, exhibits various intriguing properties depending on the twist angle of the bilayer graphene [11]. For example, flat band structures emerge close to the Fermi level in BLG superlattices when the twist angle is roughly 1.1°, which can lead to unconventional superconductivity [15,16,17,18]. It has been observed that the mobility of Bernal-stacked multilayer graphene is lower than that of monolayer graphene due to its nonlinear band dispersion [59]. On the other hand, studies have reported that the mobility of non-Bernal-stacked MLG can surpass that of monolayer graphene due to the suppression of carrier scattering caused by the substrate [60,61]. This suppression is achieved through the presence of the first layer of the turbostratic stacked MLG neighboring the substrate. In addition, it has recently been reported that dual-gated 30°-twisted bilayer graphene exhibited ultrahigh mobility up to 5 × 10^4^ cm^2^ V^−1^ s^−1^ at room temperature [62]. Thus, a recent study focused on the control of the twist angle of bilayer graphene during synthesis. 

To this end, the effects of the flow ratio of H_2_ to CH_4_ gas on the stacking orientation of the synthesized bilayer graphene on a Cu surface were investigated [38]. When the flow ratio of H_2_ to CH_4_ gas is high, the growth rate of both first layer and adlayer graphene is low, and the resulting graphene has AB stacking. On the other hand, when the ratio was relatively low, the first layer and adlayer graphene did not form AB stacking.

Cho et al. focused on the role of H_2_ in determining the twist angle of bilayer graphene during CVD [63]. Consistent with the previous reports, they revealed that a non-Bernal-stacked bilayer graphene was mainly formed when the flow rate of H_2_ was low whereas a high flow rate of H_2_ induced the formation of AB-stacked bilayer graphene. To explain the observed effects of H_2_ on the stacking, the total energy of the bilayer graphene/Cu(111) system is calculated by varying the twist angle between the graphene layers using the density functional theory. When the edge of graphene is passivated by the underlying Cu surface, which is the case of a low H_2_ flow rate, the dependence of the total energy of the system on the twist angle is mainly determined by the interaction between the second-layer graphene and underlying Cu surface. On the other hand, when the edge of the graphene is passivated by H atoms, the interaction between the second-layer graphene and Cu surface becomes weak. As a result, the interaction between the first-layer and second-layer graphene determines the total energy of the bilayer graphene/Cu system. Thus, the AB stacking becomes the most energetically favorable stacking for the bilayer graphene when the graphene edge is passivated by H atoms.

**Figure 7:**
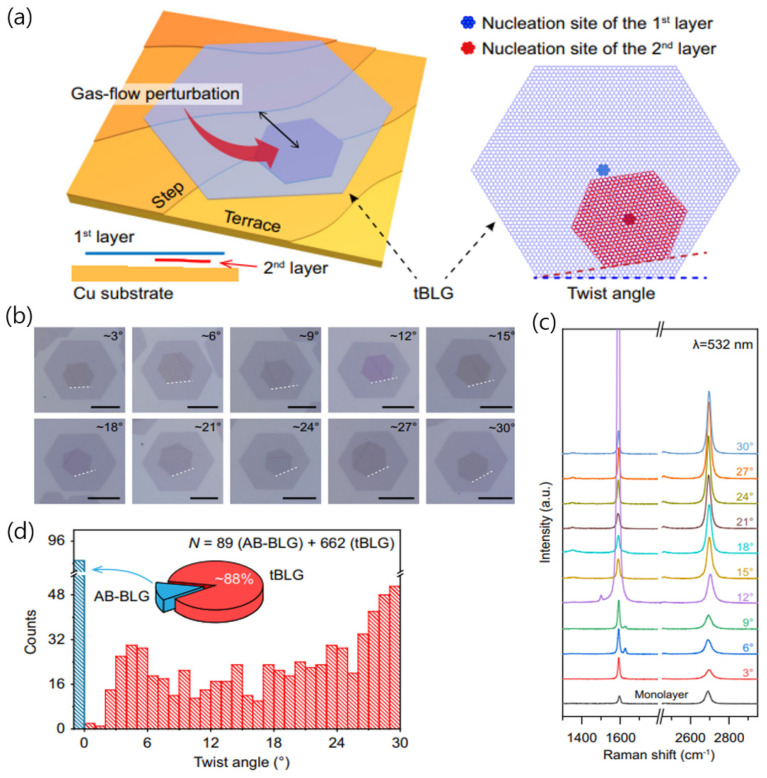
(**a**) Illustration of the hetero-site nucleation process involved in the growth of twisted bilayer graphene (tBLG) on a Cu substrate. The nucleation site for the second layer of graphene (red) is distinct from that of the first layer (blue). The lateral perspective demonstrates the subsurface growth of the second layer of graphene underneath the initial layer. Notably, the nucleation characteristics of graphene are typically influenced by the microscopic surroundings near the nucleation site, such as the presence of Cu steps. (**b**) OM images for as-grown tBLGs exhibiting twist angles of approximately 3° to 30°, incrementing by intervals of 3°. The scale bars indicate a length of 10 μm. (**c**) Raman spectra of the corresponding tBLG samples in (**b**). (**d**) Statistical analysis on the stacking order (AB stacking or non-AB stacking) and distribution of twist angles by the SAED patterns obtained from as-grown domains of the bilayer graphene. Reproduced with permission from [64]. Copyright 2021, Springer Nature.

A recent study provided a synthesis method for twisted bilayer graphene, referred to as the hetero-site nucleation strategy (Figure 7a) [64]. If the microscopic environments during the nucleation of the first and second graphene layers are the same, the resulting bilayer graphene has AB stacking configuration. However, by introducing a gas-flow perturbation to initiate the nucleation of the second layer after the first layer has formed, the environment at the time of nucleation can be different, resulting in a nucleation site distinct from the first layer (hetero-site nucleation) (Figure 7b,c). This process also creates a difference in the local environment for the graphene nuclei, including the properties of the underlying Cu surface, which enables the formation of twisted bilayer graphene. Without hetero-site nucleation, only 16% of the bilayer domains had a twisted structure, while this fraction increased to 86% when hetero-site nucleation was used (Figure 7d).

In addition to the growth conditions, the crystallographic orientation of the Cu surface is an important factor that determines the stacking orientation of BLG. Cho et al., compared the stacking orientations of BLGs grown on atomically flat (Cu(111)) and stepped Cu surfaces (Cu (311)/Cu(110)) when the other growth conditions were the same for both cases [65]. Bernal-stacked bilayer graphene was formed on the atomically flat Cu surface. However, small-angle twisted BLGs are energetically more favored on the atomically stepped Cu surface. Fine tuning of the stacking angle of BLG could be achieved through fine control of the crystallographic orientation of the Cu surface.

## 5. Summary and Perspectives

In this review, we covered the CVD method for the synthesis of multilayer graphene, discussing the growth mechanism of graphene on Ni and Cu catalysts and the challenges associated with the provision of uniformity and the control of thickness (also see Table S1, Supplementary Materials). We highlighted the use of Cu/Ni alloys as catalysts to overcome these challenges, with the concentration of Ni or the crystallinity of the Cu/Ni surface used to control the uniformity and thickness of the graphene. We also discussed alternative strategies to synthesize multilayer graphene, including the induction of a bulk diffusion of carbon atoms from the back side to the front side of Cu foils or the introduction of vacancies in early formed graphene on Cu surfaces. Additionally, we reviewed the recent research on the control of the stacking configuration of bilayer graphene during CVD synthesis, with a focus on AB-stacked and twisted bilayer graphene.

Although significant progress has been achieved in the CVD of multilayer graphene, further studies are required to fully understand its growth mechanism and achieve precise control of the thickness and uniformity. An in-depth understanding of the growth mechanism of bilayers is crucial to improve the quality of bilayers, particularly in terms of stacking orientations. Additionally, the production of high-quality graphene at low temperatures is essential for practical applications. Moreover, the development of a catalyst-free CVD method for multilayer graphene synthesis is desirable. Such an approach would eliminate the need for costly catalyst materials and simplify the overall process, including the transfer step, thus reducing production costs.

## Data Availability

No new data were created or analyzed in this study. Data sharing is not applicable to this article.

## Supplementary Materials

The following supporting information can be downloaded at: https://www.mdpi.com/article/10.3390/nano13152217/s1, Table S1. Summary of methods for synthesis of multilayer graphene.
