# Supplementary material for: Recent Understanding in the Chemical Vapor Deposition of Multilayer Graphene: Controlling Uniformity, Thickness, and Stacking Configuration
Source: Nanomaterials (Basel). 2023 Jul 30;13(15):2217. doi: 10.3390/nano13152217 (PMC10421010; doi:10.3390/nano13152217)
Supplement: Supplementary file 1 [file nanomaterials-13-02217-s001.zip › nanomaterials-2506816-supplementary.pdf]

nanomaterials

Review

Recent Understanding in Chemical Vapor Deposition of Multilayer Graphene: Controlling uniformity, Thickness, and Stacking Configuration

Hyo Chan Hong[1,†], Jeong In Ryu[1,†] and Hyo Chan Lee[1,*]

1 Department of Chemical Engineering, Myongji University, Yongin 17058, Republic of Korea
† These authors contributed equally to this work
* Correspondence: hyochan@mju.ac.kr

Table S1. Summary of methods for synthesis of multilayer graphene

Catalyst	Growth condition	No. of layers	Stacking configuration	Ref
Polycrystalline Cu/Ni film (370/20-130 nm)<br>Ni (at. %) 10.4-27.4 %	Temperature 900 ℃<br>Time 0-60 min<br>pressure 10^{-3}-10^{-4} Pa	2-5 layers	-	[1]
Single-crystal Cu/Ni(111) film (500 nm)<br>Ni (at. %) 22-25 %	Temperature 1075 ℃<br>Time 30 min	2 layer	AB and turbostratic stacking	[2]
Large-area single-crystal Cu/Ni(111) foil<br>Ni (at. %) 15.0-20.3 %	CH_4 / H_2 / Ar = 1 / 10 / 200 sccm<br>Temperature 1075 ℃<br>Pressure 40 Torr<br>Time 5 min	1-3 layers	AB stacking	[3]
Cu foil (200/0-300 nm)	CH_4 50 sccm<br>Temperature 1000 ℃<br>Time 120 min<br>Pressure 40 mTorr	1-10 layers	AB stacking	[4]
P/Cu foil	CH_4 / H_2 = 45 / 10 sccm<br>Temperature 1000 ℃<br>Time 10–80 min	3-7 layers	AB-like stacking	[5]
Cu/Ni(111) film (385/115 nm)<br>Ni (at. %) 23 %	CH_4 = 200 ppm<br>Temperature 1085 ℃<br>Time 10 min	2 layer	AB and turbostratic stacking	[6]
	CH_4 = 200 ppm<br>Temperature 1085 ℃<br>Time 10 h	2 layer	AB stacking	
Ni(111) film (500nm)	C_2H_4 7 × 10^{-6} mbar<br>Step 1 850 °C<br>Step 2 790 °C	2 layer	uniform 15° turbostratic stacking	[7]
	C_2H_4 7 × 10^{-6} mbar<br>Step 1 850 °C<br>Step 2 720 °C	2 layer	AB stacking	
Cu foil	CH_4 / H_2 = 30 / 10 sccm<br>Temperature 1000 ℃<br>Pressure 10^{-1} Torr<br>Time 10 min	2 layer	AB stacking	[8]
	CH_4 / H_2 = 30 / 100 sccm<br>Temperature 1000 ℃<br>Pressure 10^{-1} Torr<br>Time 10 min	2 layer	AB stacking<br>and AA-like or AA′ stacking	
Cu foil	CH_4 / H_2 = 400 / 0.1 sccm	1-2 layers	Mostly AB stacking (84%)	[9]
	Step 1 CH_4 / H_2 = 400 / 0.1 sccm<br>Step 2 CH_4 / H_2 = 1000 / 1.0-1.6 sccm		Mostly Turbostratic stacking (86%)	
Atomic-flat surface Cu (111) foil (0.02 mm)	CH_4 / H_2 / Ar = 20 / 250 / 100 sccm<br>Temperature 1050 ℃<br>Pressure 20 Torr	1-2 layers	AB and 30° turbostratic stacking	[10]
Atomic-stepped surface Cu (311)/(110) foil (0.02mm)			Mostly 5° turbostratic stacking	

References

1. Liu, X.; Fu, L.; Liu, N.; Gao, T.; Zhang, Y.; Liao, L.; Liu, Z. Segregation growth of graphene on Cu–Ni alloy for precise layer control. *J. Phys. Chem. C.* **2011**, *115*, 11976-11982.
2. Takesaki, Y.; Kawahara, K.; Hibino, H.; Okada, S.; Tsuji, M.; Ago, H. Highly uniform bilayer graphene on epitaxial Cu–Ni (111) alloy. *Chem. Mater.* **2016**, *28*, 4583-4592.
3. Huang, M.; Bakharev, P.V.; Wang, Z.-J.; Biswal, M.; Yang, Z.; Jin, S.; Wang, B.; Park, H.J.; Li, Y.; Qu, D. Large-area single-crystal AB-bilayer and ABA-trilayer graphene grown on a Cu/Ni (111) foil. *Nat. Nanotechnol.* **2020**, *15*, 289-295.
4. Yoo, M.S.; Lee, H.C.; Lee, S.; Lee, S.B.; Lee, N.S.; Cho, K. Chemical vapor deposition of bernal-stacked graphene on a Cu surface by breaking the carbon solubility symmetry in Cu foils. *Adv. Mater.* **2017**, *29*, 1700753.
5. Yoo, M.S.; Lee, H.C.; Lee, S.B.; Cho, K. Cu-Phosphorus Eutectic Solid Solution for Growth of Multilayer Graphene with Widely Tunable Doping. *Adv. Funct. Mater.* **2021**, *31*, 2006499.
6. Solís-Fernández, P.; Terao, Y.; Kawahara, K.; Nishiyama, W.; Uwanno, T.; Lin, Y.-C.; Yamamoto, K.; Nakashima, H.; Nagashio, K.; Hibino, H. Isothermal growth and stacking evolution in highly uniform Bernal-stacked bilayer graphene. *ACS Nano* **2020**, *14*, 6834-6844.
7. Wei, W.; Zhang, C.; Li, H.; Pan, J.; Tan, Z.; Li, Y.; Cui, Y. In Situ Growth Dynamics of Uniform Bilayer Graphene with Different Twisted Angles Following Layer-by-Layer Mode. *J. Phys. Chem. Lett.* **2022**, *13*, 11201-11207.
8. Lim, H.; Lee, H.C.; Yoo, M.S.; Cho, A.; Nguyen, N.N.; Han, J.W.; Cho, K. Effects of hydrogen on the stacking orientation of bilayer graphene grown on copper. *Chem. Mater.* **2020**, *32*, 10357-10364.
9. Sun, L.; Wang, Z.; Wang, Y.; Zhao, L.; Li, Y.; Chen, B.; Huang, S.; Zhang, S.; Wang, W.; Pei, D. Hetero-site nucleation for growing twisted bilayer graphene with a wide range of twist angles. *Nat. Commun.* **2021**, *12*, 2391.
10. Cho, H.; Park, Y.; Kim, S.; Ahn, T.; Kim, T.-H.; Choi, H.C. Specific stacking angles of bilayer graphene grown on atomic-flat and-stepped Cu surfaces. *NPJ 2D Mater. Appl.* **2020**, *4*, 35.
